# Implementation Outcomes of the National Skin Smart Campus Initiative Among Young Adults

**DOI:** 10.3390/ijerph23020166

**Published:** 2026-01-28

**Authors:** David Perez, Janessa M. Mendoza, Muriel R. Statman, Nesreen Shahrour, Ben W. Gratz, Kenneth P. Tercyak

**Affiliations:** 1 Georgetown University School of Medicine, Washington, DC 20007, USA; 2 Lombardi Comprehensive Cancer Center, Georgetown University, Washington, DC 20057, USA

**Keywords:** Skin Smart Campus, skin cancer prevention, sun safety, young adults, indoor tanning

## Abstract

Young adults often underuse ultraviolet radiation (UVR) protective strategies and engage in indoor tanning, heightening lifetime skin cancer risk. The national Skin Smart Campus (SSC) initiative encourages universities to adopt policies that reduce UVR exposure. We evaluated a 6-month SSC university campaign using an independent-samples pre-post design with surveys before (N = 230) and after (N = 267) implementation. The campaign included SSC designation, an educational webpage, targeted Instagram content, small media, and sunscreen dispensers. Following campaign launch, knowledge increased over time (t = 5.02, df = 493, *p* < 0.001), as did dispenser use (21.9% to 57.7%; χ^2^ = 64.4, *p* < 0.001). The sun safety behavior composite showed an upward trend (13.5 to 14.2, t = 1.71, df = 490, *p* = 0.09). Variance models indicated a significant time effect (F [1, 482] = 4.55, *p* = 0.03, η^2^p = 0.01; small effect), with higher sun safety associated with greater knowledge (F = 8.29, *p* = 0.004, η^2^p = 0.02; small effect) and SSC campaign awareness (F = 56.88, *p* < 0.001, η^2^p = 0.10; large effect). In multivariable regression, campaign engagement predicted higher odds of dispenser use (Odds Ratio = 3.01, 95% CI: 1.82–4.98, *p* < 0.001). Implementing SSC with environmental supports and tailored education increased knowledge, sun safety, and dispenser use, highlighting the strong influence of SSC visibility and multimodal campus-wide prevention strategies.

## 1. Introduction

Skin cancer is the most common cancer in the United States (US), with approximately one in five Americans projected to develop it by the age of 70 [1]. Over 5 million new cases are treated annually, and in 2025, 104,960 new cases of invasive melanoma—the deadliest form of skin cancer—are expected to be diagnosed [2]. Ultraviolet radiation (UVR), both from natural sun exposure and artificial sources such as indoor tanning devices, represents the leading modifiable risk factor for skin cancer [3]. Although UVR includes UVA, UVB, and UVC wavelengths, only UVA and UVB radiation can penetrate the Earth’s surface. While UVB primarily damages the outer layers of the skin and causes sunburns, UVA penetrates deeper layers, playing a significant role in photoaging, melanogenesis, and skin cancer risk [4]. Preventive behaviors such as sunscreen use, protective clothing, shade seeking, and avoiding indoor tanning are important strategies to reduce UVR exposure. However, for certain behaviors such as sunscreen use, understanding the level of protection it offers is critical. Sunscreen Sun Protection Factor (SPF) reflects how effectively a product protects the skin from UVB radiation, yet it does not account for UVA exposure [4]. To obtain full UVR protection from the sun, sunscreens that protect against both UVA and UVB, known as broad-spectrum, are essential. When used appropriately, these preventive behaviors have been shown to be highly effective in preventing skin cancer incidence. Despite this, they remain underutilized, particularly among young adults [5].

Compared to older adults, young adults are more likely to underestimate their personal skin cancer risk and engage in risky behaviors like indoor tanning and inadequate sun protection [6]. Consecutive national surveys by the American Academy of Dermatology (from 2023 to 2025) continue to demonstrate that many young adults are unaware of the dangers of UVR [7,8,9]. In their most recent survey, for example, 37% of respondents did not recognize the risks of tanning, and 57% believed in tanning myths such as the safety of a base tan [9]. Studies have further shown that UVR exposure during the teen years and early adulthood plays a key role in shaping long-term melanoma risk [6]. This risk is amplified by evidence that first exposure to tanning beds prior to age 35 can increase the risk of melanoma by 75% [10]. Early initiation of indoor tanning not only increases immediate UVR exposure, but also predicts continued use into adulthood [11,12]. Although recent epidemiologic findings indicate a decrease in indoor tanning among young adults [13], possibly balanced by the rising use of ultraviolet-free tanning products, the broad availability of tanning facilities on or near university campuses continues to expand both access to this behavior and its social normalization [14].

In addition to artificial UVR exposure, research has repeatedly shown inconsistent sun protective behaviors among young adults, placing them at significant risk of melanoma: five or more blistering sunburns between the ages of 15 and 20 may increase risk by 80% [15]. Rates of sunburn are particularly high among university students engaging in outdoor sports or recreational sunbathing, and non-White individuals as well as those of a younger age are less likely to engage in consistent sun protection [16,17]. Even among young adults aware of the risks associated with UVR exposure, a significant proportion report intermittent or minimal sunscreen use, suggesting that awareness alone is insufficient to change behavior [18]. Data from the National Health and Nutrition Examination Survey from 2013 to 2018 support these observations, showing that young adults aged 20 to 39 were less likely than those aged 40 to 59 to follow multiple protective practices, such as using sunscreen, wearing long sleeved clothing, or seeking shade [5].

Emerging research also points to deeper intra- and interpersonal mechanisms that sustain these behaviors, such as appearance-based motivations and internalized beauty ideals, as conceptualized by the Tripartite Influence Model [19]. These individual- and peer-level influences suggest that interventions targeting both norms and awareness may be particularly effective. Among such studies, for instance, Day and colleagues [19] observed that tanning among young women was mediated by cognitions of a “tanned ideal,” while men’s behavior was shaped more by peer norms. Taken together, these findings suggest that motivation, environmental accessibility, and social influence can interact to place young adults at particularly high risk for UVR overexposure. The persistence of these behaviors emphasizes the need for targeted interventions within a structured setting where young adults congregate (e.g., university campuses) that not only increase knowledge but also promote sustainable protective practices and address structural factors, such as tanning bed accessibility.

To broadly address these gaps, many skin cancer prevention interventions have been implemented for young adults, including on university campuses, with important implications for public health. In a study by Wu and colleagues [20], for example, university students were randomly assigned to one of four interventions: education alone, education plus an ultraviolet photo showing participants’ underlying skin damage from UVR exposure, education plus genetic testing (via a saliva test for *MC1R*, a gene associated with melanoma risk), or education with both an ultraviolet photo and genetic testing. Results showed that the intervention combining skin cancer prevention education with a personalized ultraviolet photo and genetic testing produced the most consistent improvements in sun safe behaviors [20]. Other intervention research, including a year-long study among more than N = 1000 young adults, showed that multimodal digital interventions delivered by social media can significantly improve sun protection behaviors, sunscreen use, and skin self-exams [21]. Collectively, these findings highlight the potential of multicomponent interventions, both in-person and digitally, for skin cancer prevention among this age group.

To help mitigate persistent UVR overexposure and tanning-related risks among university students, the National Council on Skin Cancer Prevention (NCSCP) established the Indoor Tan-Free Skin Smart Campus (SSC) initiative, which encourages universities to promote skin cancer prevention through indoor tanning bed bans, access to on-campus sunscreen dispensers, and educational programming [22]. The SSC reflects a broader national strategy outlined in the 2014 Surgeon General’s Call to Action to Prevent Skin Cancer, which recommended five key steps for individuals, institutions, and communities to decrease UVR exposure: (1) boosting sun protection in outdoor spaces, (2) informing the public about UVR risks, (3) supporting prevention-focused policies, (4) reducing indoor tanning, and (5) enhancing research and monitoring efforts [23]. Since the SCC initiative was established, over 40 universities across the nation have been designated as an SSC partner [22]. Yet, despite the growing adoption of the SSC initiative, only a single pilot study has reported on its outcomes. Among the N = 203 participants at an SSC partner campus with 10 sunscreen dispensers, 35.5% of young adults reported seeing the dispensers and 22.7% reported actual use [24]: these metrics are modest but encouraging. Although sunscreen dispenser use was generally infrequent, 91.3% reported increased sun safety awareness, pointing to the need for increased visibility of product placement in future rollouts [24].

Little remains known about the SSC initiative’s real-world implementation outcomes among young adults on university campuses. Prior study has focused on short-term, single-component interventions, leaving gaps in understanding about how multimodal, school-wide campaigns may influence both individual prevention practices and the utilization of on-campus UVR protection resources. To address this, the present study evaluated the implementation of a six-month SSC public health education campaign at a large urban university. It assessed changes in young adults’ skin cancer knowledge, awareness of the SSC initiative, adoption of sun safety behaviors, and sunscreen dispenser utilization before and after the campaign. By assessing the impact of a national initiative in a university setting, this research provides much needed insight into how health promotion efforts driven by educational institutions can influence individual prevention behaviors among young adults to reduce skin cancer risk.

## 2. Methods

### 2.1. Study Design

This research was designed as a quasi-experimental, independent-samples pre-test and post-test to evaluate the impact of a six-month SSC public health education campaign on sun safety behaviors and on-campus sunscreen dispenser use. All SSC infrastructure, including campus sunscreen dispensers, were in place three months prior to the baseline assessment period. To evaluate campaign effectiveness, cross-sectional surveys were anonymously administered before and after the campaign using the mobile-friendly platform Qualtrics (Experience Management Platform), with post-test items identical to those administered at pre-test when change over time was investigated. The pre-survey was conducted for four weeks from August to September 2024, and the post-survey was administered from March to April 2025. This timeline was selected because it aligned with the start of the university semester and covered the anticipated peak period of risk for students, which occurs during the spring semester when the full student body is present on campus. Both of the surveys and campaign took place at Georgetown University in Washington, DC.

### 2.2. Recruitment

Participants were recruited in a nonrandom manner either in-person or through a university-affiliated SSC social media account maintained by the researchers. For in-person recruitment, young adults passing through a common campus area were invited to participate at a table staffed by the research team. This sampling method was chosen for its practicality and alignment with the aim of assessing the campaign within a targeted university setting. To be eligible for the evaluation, participants were adults (age 18 years or older) and currently enrolled at the university as an undergraduate, graduate, or professional student and attending courses on the main or adjacent medical campus. To protect participant anonymity, the age of participants and their enrollment status were self-reported; however, university email accounts were required to login to the survey portal. All participants electronically consented to participating in the study prior to accessing either survey, and this study was exempted from review by the Georgetown University Institutional Review Board (STUDY00008246) in July 2024.

### 2.3. SSC Public Health Education Campaign

The SSC campaign included both digital (webpage, social media) and in-person (small media) components, as described below.

**SSC Designation:** To establish the university as an SSC partner, multiple coordinated steps were undertaken between the university’s administration and the NCSCP. The NCSCP is a coalition of public health nonprofits and government entities that support national skin cancer prevention efforts by sponsoring initiatives such as SSC and establishing the criteria universities must meet to receive formal designation. A member of the study team proposed the initiative to the university’s student health service, outlining both its significance and the requirements. To qualify, the university would have to: (1) ban indoor tanning devices in all university-owned or controlled housing and campus buildings, including those with tenants, (2) exclude businesses offering indoor tanning from university-affiliated debit card programs used by students, and (3) create an educational webpage on UVR risks and skin cancer prevention that is accessible at all times.

After supporting the university in meeting these requirements, a memorandum of understanding (MOU) was established between the NCSCP and the university. In turn, the university became eligible to receive two sunscreen dispensers at no cost, a one-year supply of sunscreen to fill them, a framed SSC certificate, and SSC signage. For its part, the university agreed to refill the sunscreen dispensers for at least two years following the first year to promote sustainability. This process was then replicated with the university’s medical school, which signed its own MOU, establishing separate agreements among the main and medical campuses due to their independent administrations. After the initiative was announced in the university’s newspaper and later communicated via email, the university’s comprehensive cancer center was engaged to anchor the program within its broader efforts of community outreach and education about skin cancer prevention.

**Webpage:** As part of the SSC initiative, an educational webpage regarding skin cancer awareness and prevention was created on the university’s student health services website. Following NCSCP recommendations, the webpage featured: (1) the university’s SSC designation, (2) the university’s commitment to keeping indoor tanning devices off campus, (3) the campus locations of the university’s sunscreen dispensers, (4) the health risks associated with UVR from the sun and indoor tanning, (5) guidance on sun safety practices, (6) information on skin cancer risk factors across skin tones, (6) criteria for melanoma detection, and (7) links to external resources for additional education.

**Social Media:** A targeted and tailored Instagram campaign was developed by analyzing the knowledge, behavior, and demographic gaps commonly reported among young adults. Pre-test data were then used to align the campaign’s guided topic selection and inform a posting schedule—prioritizing more frequent posts on topics with the greatest gaps. Posts were designed to be relevant to young adults by incorporating pop culture references to help boost engagement. Content included both educational and outreach-focused material to raise skin cancer awareness and encourage survey participation, respectively. SSC exposure was quantified using social media engagement metrics, including the number of Instagram posts, total likes, and follower counts. These metrics were collected directly from the platform, summarized over the campaign period, and are reported in detail elsewhere [25].

**Small Media:** To complement the digital component, flyers were posted across the main and medical campuses, including university residence halls, to raise awareness about the SSC campaign. Two versions of the flyer were developed, including a standard version posted throughout the campaign, and a seasonal sun safety edition during colder months. Research team members also engaged in over 85 total hours of in-person tabling at high-traffic campus locations, consisting of a health education station to raise awareness of the SSC campaign and promote young adults’ engagement through informational materials and giveaways (e.g., UVR color changing wristbands, SPF 50 lip balm).

### 2.4. Sunscreen Dispenser Availability

At campaign launch, four sunscreen dispensers were available on campus, with two located on the university’s main campus and the other two at the medical campus. The locations were selected for their high student foot traffic (e.g., dining court) and were also positioned near building exits to encourage and facilitate sun protection before going outdoors. In month five of the campaign, three additional dispensers were installed, bringing the total to seven. These three were installed at the university’s athletic centers to expand the reach of the intervention in response to young adults’ interest.

### 2.5. SSC Campaign Evaluation Measures

**Demographics:** Following electronic informed consent, participants in the evaluation sample reported on their age (in years), sex, race, ethnicity, and program of study (e.g., undergraduate, medical, or nursing student [undergraduate or graduate], other).

**Covariates:** Skin cancer-related knowledge was evaluated using four true/false questions, including: (1) “Skin cancer is the most common cancer in the US” (correct: true), (2) “Skin cancer only occurs in Type I to Type V skin tones” (correct: false), (3) “Tanning beds do not increase the risk of developing melanoma if they are used before the age of 35” (correct: false), and (4) “Tanning beds emit more UVR than the sun” (correct: true). Each correct knowledge item received an individual score of 1, and then a composite score was calculated by summing across all four items (range = 0–4); higher scores indicated greater knowledge. This four-item knowledge scale was intended to be face-valid and was adapted from skin cancer facts and prevention information provided by the Skin Cancer Foundation, with items reflecting core domains aligned with the public health education campaign’s educational content [26]. Awareness of the SSC initiative was measured utilizing a single binary (yes/no) question assessing whether participants were aware of the university’s SSC designation.

**Dependent variables:** Sun safety behaviors were evaluated using seven items on a 5-point scale (0 = never, 1 = rarely, 2 = sometimes, 3 = often, 4 = always), drawn from Glanz and colleagues [27,28]. Participants reported on: (1) using sunscreen with SPF 30 or higher, (2) reapplying sunscreen after two hours, sweating, or swimming, (3) wearing a hat, (4) using protective clothing that covers the arms and legs, (5) wearing sunglasses, (6) seeking shade when outdoors, and (7) checking the ultraviolet index before outdoor activity. Items were analyzed individually and then summed to create a composite score (range = 0–28) that reflected overall engagement in sun protective behaviors: higher scores indicated greater engagement in sun safety. Acknowledging the composite score is a sum of ordinal items, we assessed normality prior to analysis, and the data were symmetric with a kurtosis of –0.48, indicating approximate normality. We also examined the combined internal consistency reliability of the scale, and it was adequate (0.70 overall, 0.57 at pre-test, 0.83 at post-test). Sunscreen dispenser use was measured by a single binary (yes/no) item regarding whether young adults had ever used an on-campus sunscreen dispenser.

### 2.6. Analysis

Prior to study initiation, statistical power was estimated using G*Power version 3.1 for an independent-samples pre–post design. Assuming approximately 225 participants at pre-test and post-test, a two-tailed α of 0.05, and equal group sizes, the study had 80% power to detect small mean differences of approximately Cohen’s d = 0.27 (and 90% power for d ≈ 0.31) for continuous outcomes. For two-group omnibus models, the available sample provided ≥80% power to detect small effects (Cohen’s f = 0.13; partial η^2^ ≈ 0.02), which aligned with expected small health education campaign effects on behavioral composites. For binary outcomes, an increase from ~20% to ≥35% yielded >80% power, with substantially higher power for larger changes. Collectively, these estimates indicated adequate power to detect the anticipated small-to-moderate campaign effects across the outcomes of interest.

To address missing data, all variables in the dataset were checked for sporadic missingness (<10%), and no data imputation methods were utilized for case analysis (list-wise deletion within the models was used). Descriptive statistics were computed to summarize participant demographics, skin cancer-related knowledge, SSC awareness, tanning bed use, sun safety behavior, and sunscreen dispenser use before and after the campaign. Bivariate tests (*t*-tests and chi-square tests) were used to guide stepwise selection of the covariates associated with the dependent variables for inclusion in subsequent multivariate analyses (Supplemental Tables S1 and S2). Analysis of covariance (ANCOVA) was conducted to compare sun safety behavior between pre- and post-survey groups, and a logistic regression was used to examine predictors of sunscreen dispenser use, each adjusting for relevant confounders. All statistical analyses were conducted using Jamovi version 2.7.6.

## 3. Results

### 3.1. Participant Characteristics

A total of N = 230 participants completed the pre-test survey and N = 267 completed the post-test survey and were included in the evaluation samples (Table 1). At pre-test, the mean age of participants was 23.7 years (standard deviation (SD) = 3.1) and 23.3 years (SD = 3.0) at post-test. Most participants identified as female (69.6% pre- vs. 65.9% post-), White (34.3% pre- vs. 45.3% post-), and non-Hispanic (92.2% pre- vs. 90.6% post-), with a mixed composition of undergraduate (%) and graduate (%) students (Table 1).

### 3.2. Skin Cancer-Related Knowledge

Knowledge of skin cancer prevention significantly improved (t = 5.02, df = 493, *p* < 0.001, Table 2) following the campaign. Correct identification of skin cancer as the most common cancer in the US increased from 65.8% pre-test to 82.0% post-test (χ^2^ = 17.1, df = 1, *p* < 0.001, Table 2). Additionally, recognition that skin cancer occurs across all skin tones rose from 93.0% to 96.6% (χ^2^ = 3.41, df = 1, *p* = 0.07, Table 2). Lastly, awareness that tanning beds increase melanoma risk improved from 93.9% to 97.8% (χ^2^ = 4.81, df = 1, *p* = 0.03), and knowledge that tanning beds emit more UVR than the sun increased from 81.1% to 90.3% (χ^2^ = 8.53, df = 1, *p* = 0.003, Table 2).

### 3.3. Sun Safety Behaviors

From pre- to post-test, the mean sun safety behavior composite score slightly increased from 13.5 to 14.2 (max = 28.0), approaching conventional significance in unadjusted analyses (t = 1.71, df = 490, *p* = 0.09, Table 3). Improvements were noted in several individual sun safety behavior components, including use of SPF 30+ sunscreen (always: 24.8% pre- vs. 44.9% -post), hat use (often/always: 20.8% vs. 32.6%), protective clothing (often/always: 28.0% vs. 42.3%), and sunglasses use (often/always: 40.3% vs. 47.6%, Table 3). In race- and age-adjusted ANCOVA models, comparing the independent pre- and post-survey samples, sun safety behaviors showed a significant time effect (pre- vs. post-) (F [1, 482] = 4.55, *p* = 0.03, η^2^p = 0.01; small effect), and were also significantly associated with skin cancer-related knowledge (F [1, 482] = 8.29, *p* = 0.004, η^2^p = 0.02; small effect) and SSC awareness (F [1, 482] = 56.88, *p* < 0.001, η^2^p = 0.10; large effect, Table 4).

### 3.4. Sunscreen Dispenser Use

Reported use of on-campus sunscreen dispensers improved from 21.9% at pre-test to 57.7% at post-test, representing an absolute increase of 35.8% (χ^2^ = 64.4, df = 1, *p* < 0.001, Table 5). Logistic regression further indicated that dispenser use was significantly predicted by group (post- vs. pre-: odds ratio (OR) = 3.01, 95% confidence interval (CI) = 1.82–4.98, *p* < 0.001, Table 6). Additionally, White participants were significantly more likely to use the dispenser compared to non-White participants (OR = 1.83, 95% CI = 1.15–2.91, *p* = 0.011), and older young adults were more likely to use the dispenser, with each one-year increase in age associated with a 13% increase in the odds of use (OR = 1.13, 95% CI = 1.04–1.22, *p* = 0.002, Table 6). Participants who were aware of the SSC initiative also showed over eight times greater odds of use compared to those unaware (OR = 8.01, 95% CI = 4.89–13.10, *p* < 0.001, Table 6).

## 4. Discussion

This study evaluated the implementation outcomes of the national SSC initiative at a large urban university, focusing on changes in skin cancer knowledge, sun safety behaviors, and sunscreen dispenser utilization among young adults. The findings show that the 6-month campaign led to notable improvements in young adults’ knowledge, awareness, and engagement in protective behaviors, suggesting that university-based interventions with multimodal components can effectively encourage skin cancer prevention practices among this high-risk group.

Overall, participants showed notable improvements in knowledge about skin cancer and awareness of the SSC initiative after the campaign. Consistent with previous studies that emphasize knowledge as a key factor influencing protective behaviors [20,27], greater knowledge was strongly associated with increased sun safety behaviors. While improvements in skin cancer-related knowledge were statistically significant, the relatively small size of the effect for sun safety behaviors suggests only a modest shift in prevention over time. In particular, the nonsignificant effect of knowledge on sunscreen dispenser use may indicate that certain preventive behaviors may be less responsive to knowledge-based interventions alone, consistent with prior research [29,30]. Despite this, these improvements offer insight regarding the potential benefits of similar campaigns, and particularly if conducted over longer periods and combined with other strategies (e.g., individual risk feedback). Additionally, being aware of the SSC initiative reflected a large effect and was a strong predictor of both engagement in sun safety behaviors and use of sunscreen dispensers, highlighting the critical role of campaign visibility and message reach in health behavior participation.

The most notable improvement observed was in sunscreen dispenser utilization, which increased by nearly 36 percentage points from pre- to post-test. Compared to Myers and colleagues’ SSC pilot [24], which reported a relatively low usage rate, the present study’s more than twofold increase in dispenser utilization may reflect the synergistic effects of educational reinforcement through digital and small media channels, the strategic placement of additional dispensers at high-traffic locations, and the social normalization of sun protection behaviors on campus. The observed link between dispenser use and higher overall sun safety suggests that on-campus resources may serve as both a behavioral cue and a facilitator of broader protective practices.

The positive changes in sun-protective behaviors align with earlier research from campus-based and digital prevention programs aimed at young adults [20,21,31]. Comparable interventions that combine education with environmental support have demonstrated improvements in sunscreen use and self-reported sun protection [32]. The SSC initiative, however, builds on these approaches by integrating prevention efforts directly into university infrastructure, focusing on sustainability through institutional policy changes and ongoing resource management. The observed increases in knowledge and behavior change among diverse young adults highlight SSC’s potential as a scalable method for promoting prevention at the population level, consistent with the 2014 Surgeon General’s Call to Action to Prevent Skin Cancer [23].

The demographic trends identified also merit particular attention. White and older young adults were significantly more likely to use sunscreen dispensers, reflecting previous findings of racial and age disparities in sun protection [17,33]. Although knowledge improved across all groups, these differences indicate that additional tailoring may be needed to boost engagement among younger subgroups and those from racially and ethnically diverse backgrounds. Future SSC campaigns should focus on culturally tailored messaging, strategically place dispensers in areas frequented by underrepresented groups, and collaborate with affinity organizations to amplify educational outreach. Including testimonials or personal stories could further enhance engagement by making the risks more relatable, an approach supported by prior interventions that showed greater behavioral impact when education is paired with personalized risk information [20]. Additionally, since older young adults were more likely to report using dispensers, younger subgroups represent a key target for communication efforts, especially via preferred platforms like YouTube, Instagram, and TikTok [34].

The success of the SSC model in this context highlights the importance of combining education with environmental and policy support. Its multicomponent design, integrating a social media campaign, in-person engagement, and physical sunscreen availability, appears to have increased the intervention’s salience and sustainability. Crucially, embedding the campaign within existing university systems (such as student health services and cancer center partnerships) likely facilitated its adoption and practicality. Future SSC implementations could benefit from incorporating monitoring systems to track dispenser refills, digital analytics, and longer-term follow-up to assess whether behavior changes are maintained beyond the initial campaign.

Several limitations should be noted. First, this study used a quasi-experimental, non-random, independent-samples design, which limits causal inference and introduces the potential for selection bias. Although improvements were seen, differences in the composition of pre- and post-test samples may partly explain the changes observed. Second, reliance on self-reported data introduces the possibility of recall bias and social desirability bias. For example, since the study team does not have access to permanent product data about dispenser refill frequency, sunscreen dispenser use, like all other sun safety behaviors, should be interpreted within the limitations of self-reported data. Third, because recruitment and enrollment occurred through open and voluntary outreach steps, such that participants who engaged with the survey either completed it or opted not to, we were unable to determine response rates, limiting our assessment of sampling bias. Fourth, since the internal consistency of the sun safety behavior composite in the pre-survey was below the commonly accepted threshold, the lower reliability at baseline may have introduced measurement error and should be taken into account when considering pre-survey estimates. Although the composite is expected to be unidimensional, greater heterogeneity at baseline may have contributed to the lower internal consistency. Fifth, while adding new sunscreen dispensers during the campaign likely improved access, it complicates attributing behavior changes solely to educational efforts. Sixth, given the higher proportion of White and undergraduate students at post-test, it is possible that some observed effects may reflect differences in sample composition rather than the campaign. Seventh, as level of SPF protection alone does not guarantee full protection against the risk of skin cancer since it does not capture whether the SPF used protected against UVA and UVB radiation or only UVB protection, use of “SPF ≥ 30” may not fully reflect skin cancer risk protection due to residual UVA exposure. Future studies should specifically ask about the use of broad-spectrum (UVA and UVB protective) sunscreen with an adequate SPF value. Eighth, survey items were largely self-developed without formal validation, potentially limiting the reliability of these findings. Finally, since the study was conducted at a single urban university, its findings may not be generalizable to other campus settings.

Despite these limitations, the findings offer valuable implementation insights to the expanding research on skin cancer prevention among young adults. The results endorse the SSC initiative as a practical and effective approach for promoting sun-safe behaviors within academic settings. Future studies should explore the long-term sustainability of these behaviors, evaluate the cost-effectiveness of sunscreen dispenser programs, and investigate how SSC efforts can be integrated with digital tools for tracking behavior to maximize their impact. Moreover, extending the initiative to a wider range of campuses, including those with more diverse student populations and different climates, could help develop equitable strategies for broader implementation.

## 5. Conclusions

The local implementation of the national SSC initiative successfully enhanced young adults’ knowledge, awareness, and participation in sun safety practices at a university. By combining public health messaging through social media, environmental changes, and institutional support, SSC campaigns offer a promising and sustainable method for advancing skin cancer prevention on university campuses across the country. Ongoing investment in these initiatives, along with focused efforts to engage underrepresented groups, may have the potential to deliver lasting public health benefits across the lifespan.

## Figures and Tables

**Table 1:** Demographics.

	Pre-Test	Post-Test
**Variable**	N = 230	N = 267
**Age, M (SD)**	23.7 (3.1)	23.3 (3.0)
**Sex, N**		
Female	160 (69.6%)	176 (65.9%)
Male	68 (29.6%)	91 (34.1%)
Non-Binary	2 (0.9%)	0 (0%)
**Race, N**		
White	79 (34.3%)	121 (45.3%)
Black/African American	21 (9.1%)	17 (6.4%)
Asian or Pacific Islander	76 (33.0%)	80 (30.0%)
Middle Eastern	22 (9.6%)	22 (8.2%)
Mixed Race	30 (13.0%)	23 (8.6%)
Other	2 (0.9%)	4 (1.5%)
**Ethnicity, N**		
Non-Hispanic	212 (92.2%)	242 (90.6%)
Hispanic	18 (7.8%)	25 (9.4%)
**Student type**		
Medical	150 (65.2%)	146 (54.7%)
Nursing (undergraduate or graduate)	2 (0.9%)	2 (0.7%)
Undergraduate	56 (24.3%)	112 (41.9%)
Other	22 (9.6%)	7 (2.6%)

**Table 2:** Covariates.

Variable	Pre-Test	Post-Test	Test Statistic	df	*p*
**Skin cancer-related knowledge, M (SD)**	3.34 (0.82)	3.67 (0.64)	t = 5.02	493	<0.001
Skin cancer is the most common cancer in the US.			χ^2^ = 17.1	1	<0.001
True (Correct)	150 (65.8%)	219 (82%)			
False	78 (34.2%)	48 (18%)			
Skin cancer only occurs in Type I to Type V skin tones.			χ^2^ = 3.41	1	0.065
True	16 (7%)	9 (3.4%)			
False (Correct)	212 (93%)	258 (96.6%)			
Tanning beds do not increase the risk of developing melanoma if they are used before the age of 35.			χ^2^ = 4.81	1	0.028
True	14 (6.1%)	6 (2.2%)			
False (Correct)	214 (93.9%)	261 (97.8%)			
Tanning beds emit more UVR than the sun.			χ^2^ = 8.53	1	0.003
True (Correct)	185 (81.1%)	241 (90.3%)			
False	43 (18.9%)	26 (9.7%)			
**SSC Awareness**			χ^2^ = 64.4	1	<0.001
Yes	55 (24.6%)	178 (66.7%)			
No	169 (75.4%)	89 (33.3%)			

**Table 3:** Sun Safety Behaviors.

In the Past Month While Outside, How Often Did You…	Pre-Test (N [%])	Mean (±SD)	Post-Test (N [%])	Mean (±SD)	Statistic	df	*p*
**Wear SPF of 30 or greater?**		2.7 (1.1)		2.7 (1.4)	t = 0.51	491	0.61
Never = 0	14 (6.2%)		28 (10.5%)				
Rarely = 1	20 (8.8%)		35 (13.1%)				
Sometimes = 2	51 (22.6%)		41 (15.4%)				
Often = 3	85 (37.6%)		43 (16.1%)				
Always = 4	56 (24.8%)		120 (44.9%)				
**Reapply sunscreen after being outside** **for 2 h, sweating or in** **the water?**		1.4 (1.1)		1.5 (1.1)	t = 0.84	491	0.40
Never = 0	54 (23.9%)		66 (24.7%)				
Rarely = 1	67 (29.6%)		57 (21.3%)				
Sometimes = 2	64 (28.3%)		85 (31.8%)				
Often = 3	32 (14.2%)		54 (20.2%)				
Always = 4	9 (4%)		5 (1.9%)				
**Wear a hat?**		1.5 (1.1)		1.7 (1.2)	t = 2.53	491	0.01
Never = 0	58 (25.7%)		60 (22.5%)				
Rarely = 1	53 (23.5%)		40 (15%)				
Sometimes = 2	68 (30.1%)		80 (30%)				
Often = 3	42 (18.6%)		82 (30.7%)				
Always = 4	5 (2.2%)		5 (1.9%)				
**Wear clothes that covered your arms and legs?**		2.0 (1.0)		2.3 (0.9)	t = 4.16	490	<0.001
Never = 0	13 (5.8%)		12 (4.5%)				
Rarely = 1	64 (28.4%)		26 (9.7%)				
Sometimes = 2	85 (37.8%)		116 (43.4%)				
Often = 3	47 (20.9%)		93 (34.8%)				
Always = 4	16 (7.1%)		20 (7.5%)				
**Wear sunglasses?**		2.1 (1.1)		2.0 (1.2)	t = 0.62	491	0.53
Never = 0	27 (11.9%)		38 (14.2%)				
Rarely = 1	31 (13.7%)		53 (19.9%)				
Sometimes = 2	77 (34.1%)		49 (18.4%)				
Often = 3	73 (32.3%)		114 (42.7%)				
Always = 4	18 (8%)		13 (4.9%)				
**Shade to avoid the sun?**		2.5 (0.9)		2.4 (1.0)	t = 0.70	490	0.48
Never = 0	7 (3.1%)		13 (4.9%)				
Rarely = 1	21 (9.3%)		22 (8.2%)				
Sometimes = 2	79 (35.1%)		97 (36.3%)				
Often = 3	88 (39.1%)		104 (39%)				
Always = 4	30 (13.3%)		31 (11.6%)				
**Check the UV index?**		1.3 (1.3)		1.4 (1.1)	t = 1.09	490	0.28
Never = 0	83 (36.9%)		72 (27%)				
Rarely = 1	56 (24.9%)		55 (20.6%)				
Sometimes = 2	33 (14.7%)		100 (37.5%)				
Often = 3	35 (15.6%)		28 (10.5%)				
Always = 4	18 (8%)		12 (4.5%)				
**Total (max = 28.0)**		13.5 (4.1)		14.2 (5.6)	t = 1.71	490	0.09

**Table 4:** Sun Safety Behaviors ANCOVA.

Subcategory	Sum of Squares	df	Mean Square	F	*p*	n^2^p
**Overall model**	1711.10	6	285.19	19.48	<0.001	
Age *	133.40	1	133.40	6.66	0.010	0.014
**Race**	180.53	1	180.53	9.01	0.003	0.018
**Ethnicity**	0.002	1	0.002	1.32 × 10^−4^	0.991	0.000
**SSC Awareness**	1139.92	1	1139.92	56.88	<0.001	0.105
Knowledge *	166.08	1	166.08	8.29	0.004	0.017
**Group**	91.14	1	91.14	4.55	0.033	0.009

* Covariates.

**Table 5:** Campus Sunscreen Dispenser Use.

Question	Pre-Test (N [%])	Post-Test (N [%])	Mean	Statistic	df	*p*
**Dispenser use**				χ^2^ = 64.4	1	<0.001
Yes	49 (21.9%)	154 (57.7%)				
No	175 (78.1%)	113 (42.3%)				

**Table 6:** Campus Sunscreen Dispenser Use Logistic Regression.

						95% Confidence Interval	
Predictor	Estimate	SE	Z	*p*	Odds Ratio	Lower	Upper
**Age**	0.122	0.0402	3.038	0.002	1.13	1.04	1.22
**Sex** Male (Ref.)—Female	0.237	0.2489	0.953	0.34	1.27	0.78	2.07
**Race** White (Ref.)—Non-White	0.603	0.2364	2.553	0.01	1.83	1.15	2.91
**Ethnicity** Non-Hispanic (Ref.)—Hispanic	−0.285	0.3926	−0.727	0.47	0.75	0.35	1.62
**SSC Awareness** Yes (Ref.)—No	2.080	0.2511	8.284	<0.001	8.01	4.89	13.10
**Knowledge**	−0.129	0.1685	−0.766	0.44	0.88	0.63	1.22
**Group** Pre-test (Ref.)—Post-Test	1.102	0.2572	4.283	<0.001	3.01	1.82	4.98

## Data Availability

Data for this study are not available due to its sensitive nature.

## Supplementary Materials

The following supporting information can be downloaded at: https://www.mdpi.com/article/10.3390/ijerph23020166/s1, Table S1: Sun-safety Behaviors Bivariate Analyses; Table S2: Dispenser Use Bivariate Analyses.

## Abbreviations

United States (US), ultraviolet radiation (UVR), National Council on Skin Cancer Prevention (NCSCP), Indoor Tan-Free Skin Smart Campus (SSC), memorandum of understanding (MOU), sun protection factor (SPF), analysis of covariance (ANCOVA), standard deviation (SD), odds ratio (OR), confidence interval (CI), Georgetown University (GU).
