# Supplementary material for: Implementation Outcomes of the National Skin Smart Campus Initiative Among Young Adults
Source: Int J Environ Res Public Health. 2026 Jan 28;23(2):166. doi: 10.3390/ijerph23020166 (PMC12941029; doi:10.3390/ijerph23020166)
Supplement: Supplementary file 1 [file ijerph-23-00166-s001.zip › ijerph-4037649-Supplementary materials.pdf]

Supplemental Table S1: Sun-safety Behaviors Bivariate Analyses

Independent Variable	Low*	High**	Statistic	Value	df	p
Pre-test						
**Age**			t-test	1.08	223	0.283
**Sex**			χ^2	0.696	1	0.404
Female	121	38				
Male	52	12				
**Race**			χ^2	3.00	1	0.083
White	54	22				
Non-White	121	28				
**Ethnicity**			χ^2	0.550	1	0.458
Non-Hispanic	163	45				
Hispanic	12	5				
**SSC Awareness**			χ^2	2.16	1	0.142
Yes	39	16				
No	135	33				
**Knowledge**			t-test	0.845	223	0.399
Post-test						
**Age**			t-test	5.91	265	<0.001
**Sex**			χ^2	1.17	1	0.279
Female	105	71				
Male	48	43				
**Race**			χ^2	6.60	1	0.010
White	59	62				
Non-White	94	52				
**Ethnicity**			χ^2	5.12	1	0.024
Non-Hispanic	144	98				
Hispanic	9	16				
**SSC Awareness**			χ^2	84.4	1	<0.001
Yes	67	111				
No	86	3				
**Knowledge**			t-test	2.14	265	0.034

*Sun-safety behavior score between 0-16

**Sun-safety behavior score between 17-28

Supplemental Table S2: Dispenser Use Bivariate Analyses

Independent Variable	No	Yes	Statistic	Value	df	p
Pre-test						
**Age**			t-test	2.11	222	0.036
**Sex**			χ^2	0.003	1	0.953
Female	124	34				
Male	50	14				
**Race**			χ^2	4.74	1	0.030
White	53	23				
Non-White	122	26				
**Ethnicity**			χ^2	0.611	1	0.434
Non-Hispanic	163	44				
Hispanic	12	5				
**SSC Awareness**			χ^2	20.2	1	<0.001
Yes	31	24				
No	144	25				
**Knowledge**			t-test	-2.09	222	0.038
Post-test						
**Age**			t-test	6.07	265	<0.001
**Sex**			χ^2	6.18	1	0.013
Female	84	92				
Male	29	62				
**Race**			χ^2	6.45	1	0.011
White	41	80				
Non-White	72	74				
**Ethnicity**			χ^2	3.79	1	0.051
Non-Hispanic	107	135				
Hispanic	6	19				
**SSC Awareness**			χ^2	96.2	1	<0.001
Yes	38	140				
No	75	14				
**Knowledge**			t-test	1.42	265	0.157
